# Attitudes of People With Chronic Musculoskeletal Disorders Towards Telerehabilitation: A Cross-Sectional Survey

**DOI:** 10.7759/cureus.87012

**Published:** 2025-06-30

**Authors:** Evgenia Tsolakou, Georgios Gioftsos, Eirini Grammatopoulou, George A Koumantakis, Stefanos Karanasios, Maria Moutzouri

**Affiliations:** 1 Department of Physiotherapy, University of West Attica, Athens, GRC; 2 Laboratory of Advanced Physiotherapy, Department of Physiotherapy, School of Health and Care Sciences, University of West Attica, Athens, GRC

**Keywords:** attitudes, chronic musculoskeletal disorders, pain management, physiotherapy, survey, tele-physiotherapy, telerehabilitation

## Abstract

Introduction

Chronic musculoskeletal disorders have a significant impact on morbidity, daily functioning, and quality of life. They represent a key priority for intervention, particularly in the context of an aging population and increased life expectancy. Recently, telerehabilitation has been shown to be effective; however, the attitudes of this clinical population toward telerehabilitation have not yet been thoroughly explored in the literature. The aim of this study was to investigate the knowledge, beliefs, and willingness of people with chronic musculoskeletal disorders in Greece to use telerehabilitation.

Materials and methods

In this cross-sectional study, 160 individuals (95 women and 65 men) with a mean age of 44.1 years and diagnosed with chronic musculoskeletal problems completed a survey. The sample included participants with low back pain (56, 36.9%), overuse tendinopathies (30, 18.8%), neck pain (34, 21.3%), arthritis (14, 8.8%), and upper back pain (3, 1.9%). Participants were recruited from physiotherapy clinics in Athens. The study protocol was approved by the Ethical Committee of the University of West Attica, Greece. The survey questionnaire included 26 items (16 general and 10 telerehabilitation-specific) covering demographic background, familiarization with technology, overall perceptions, willingness to follow telerehabilitation, and preferred mode of delivery.

Results

The survey was first pilot-tested, and relevant modifications were made. The final version demonstrated acceptable internal consistency (Cronbach’s alpha = 0.648). Principal Components Analysis (PCA) revealed a three-factor solution, familiarization with technology, preferred mode of telerehabilitation, and willingness to attend telerehabilitation, with eigenvalues of 2.19, 1.98, and 1.22 respectively, explaining 53.97% of total variance. Overall, fewer than half of the participants (59, 36.9%) reported willingness to follow telerehabilitation. The preferred mode of delivery was video-based sessions. Their perspective towards telerehabilitation appeared to be related to seeking information about their problem online (X^2^_(4, N=160)= _11.1, p=0.03) and technological familiarity (Likelihood Ratio, p=0.048) but not with the specific type of musculoskeletal condition (X^2^_(16, N=160)_=12.7, p=ns).

Conclusion

Telerehabilitation, which leverages technology to deliver rehabilitation services remotely, is indeed seeing its culture constructed and integrated into healthcare systems. However, this survey highlights barriers based on patient attitudes, as less than half of respondents were receptive to the approach. Higher education levels, greater familiarity with technology, and a tendency to seek medical information online were associated with a greater willingness to engage in telerehabilitation. Further research involving larger samples and populations from rural areas across Greece is needed to assess the generalizability of these findings, particularly in regions where health equity is challenged by limited access and technological disparities.

## Introduction

Chronic musculoskeletal disorders account for approximately 21.3% of global morbidities [1]. Over a lifetime, more than 25% of the worldwide population experiences these chronic disorders, including low back pain, neck pain, osteoporosis, arthritis, and degenerative conditions caused by injuries or overuse, such as tendinopathies [2,3]. Low levels of long-term adherence to exercise among patients with chronic musculoskeletal conditions negatively impact treatment effectiveness, increase symptom recurrence, lead to patient neglect, and reduce productivity [4]. As a result, individuals with chronic conditions often miss scheduled sessions, leading to a vicious cycle of psychosomatic complaints, depression, anxiety, low self-confidence, avoidance of exercise, and deterioration of physical fitness [5]. For this reason, a convincing digital approach that acknowledges the long-term impact of chronic disorders is essential to support prevention and control efforts and help maintain a healthy lifestyle throughout life [6]. In this context, it is important for patients to select interventions that are both cost-effective and capable of delivering substantial health benefits with minimal resource investment (e.g., time, space, cost, transportation).

Telerehabilitation has been identified as a cost-effective therapeutic approach to promote self-management and reduce the need for continuous clinical oversight in patients with chronic musculoskeletal disorders [7-9]. It addresses key barriers such as cost and transportation, especially for individuals in rural or remote areas [6,10], as well as time constraints, since sessions can be completed at the patient’s convenience, from home or a community setting, without the need for sophisticated or expensive equipment. However, due to aging, limited access to technology, or a preference for face-to-face sessions, some patients may hesitate to adopt telerehabilitation, even though various modalities are available (e.g., telephone-based, video-based, web-based, sensor-based) [11]. Özden F et al. [12] found that an 8-week telerehabilitation protocol using exercise videos and therapist communication software significantly improved pain, functionality, kinesiophobia, motivation, and satisfaction in patients with chronic low back pain. Interestingly, the telerehabilitation group reported greater improvements across all parameters compared with a conventional home-based rehabilitation group. Brigo E et al. [13] reported that, particularly during the pandemic, telerehabilitation was a feasible, safe, and effective method for maintaining high-quality care and enhancing home-based self-management for chronic conditions. Repeated sessions and video-based exercise programs may further encourage long-term adherence and enhance patients’ motivation and self-management skills [14]. Nevertheless, barriers such as low self-efficacy, kinesiophobia, and poor compliance continue to limit the adoption of telerehabilitation [15].

Patient-centeredness has been reported as one of the six dimensions of quality-valued healthcare [16]. Patients’ attitudes represent a central axis in the perception of social states, such as personality, that guide a person’s behavior in interactions with others within a psychosocial environment [17]. A person’s attitude is shaped by their perceptions, social influences, and previous experiences. The successful development of a telehealth solution requires a deep understanding of patients’ needs and perceptions, as well as the involvement of relevant stakeholders. Previous studies involving orthopedic, geriatric, or neurological patients have explored perceptions and experiences with telerehabilitation, which have generally been reported as satisfactory [18-20]. However, the attitudes and willingness of patients with chronic musculoskeletal disorders, particularly those with no prior experience of telerehabilitation, have not been previously investigated. This represents the novelty of the current study.

Therefore, the aim of the present study is to investigate the attitudes of patients with chronic musculoskeletal disorders towards the telerehabilitation approach. The sub-objectives are to explore: (1) the preferred modes of telerehabilitation that patients would be more willing to follow; (2) the perceived needs that telerehabilitation aims to fulfill; and (3) potential facilitators and barriers for this clinical population in engaging with such a program. It is our hope that the findings of this study will inform the development of a telerehabilitation platform that helps chronic patients remain connected to treatment in a convenient manner, enhances self-management, reduces their reservations, and ultimately supports clinicians in delivering effective care.

Part of the findings from this study has been previously presented as a poster: Moutzouri M, Tsolakou E, Koumantankis G, Karanasios S, Gioftsos G. The perspectives of people with chronic musculoskeletal problems on telerehabilitation (PO 14652), IFOMPT Congress, Basel, 2024.

## Materials and methods

The current study is a cross-sectional survey utilizing a closed-ended questionnaire. Ethical approval was obtained from the West Attica University Research Ethics Committee, Athens, Greece (21486/03-03-2023).

Participants

Participants were eligible to participate in the survey if they were between 18-65 years old and presented with common chronic musculoskeletal conditions (pain >3 months), including low back pain, neck pain, osteoarthritis, osteoporosis, and overuse syndromes. Individuals with comorbidities such as neurological disorders or severe cognitive impairments were excluded.

Participants were recruited through systematic sampling (every nth arriving patient) from outpatient clinics in the Attica region, from September 2022 to May 2023. Consenting individuals who met the inclusion criteria completed the questionnaire in paper format to avoid potential bias that may arise with online completion by participants more familiar with technology. A specialized musculoskeletal physiotherapist (SK), with over 15 years of experience, conducted the clinical assessments to confirm the diagnosis of chronic musculoskeletal disorder.

Sample number

To meet methodological requirements, a minimum of 130 participants was needed, based on a recommended minimum of five participants per item (26 items) for scale validation [21]. To account for possible dropouts or missing data, an additional 25% was added to the sample, resulting in 170 participants being approached for the study.

Questionnaire

The questions were developed through a focus group involving professionals from various specialties of physiotherapy in the field of rehabilitation, with the assistance of a senior researcher. The questionnaire construction followed the recommended guidelines of Roopa S and Satya RM [19]. First, the aims of the study were clearly defined and carefully translated into question content. Second, an expert in questionnaire construction and psychometric assessment (a senior researcher) was consulted to evaluate the design and phrasing of the questions in terms of clarity, comprehension, language, and potential bias [22]. Careful attention was paid to the type, content, and order of the questions included. Relevant modifications were made accordingly to ensure the questionnaire accurately reflected the concept of the survey. Third, a draft questionnaire was piloted with 30 participants to assess the clarity of language, understanding, and accuracy in expressing four key themes: perceptions, needs, facilitators, and barriers. Discussions during the pilot phase verified the accurate expression of social perceptions and identified any unclear questions or those that should be merged or removed. The final version of the questionnaire was completed and used for data collection. A Likert-type scale was employed as a psychometric instrument to measure respondents’ attitudes, with symmetrical response options structured on a five-point scale ranging from “Strongly Disagree” to “Strongly Agree.” The average time required to complete the questionnaire was approximately 5 to 10 minutes.

Appendix 1 presents the questionnaire layout, and Appendix 2 provides the complete survey questionnaire. As shown, the questionnaire consisted of two sections: (a) a general preliminary section with 16 items, covering demographics, background, knowledge of the musculoskeletal disorder, and familiarization with technology; and (b) a specific section with 10 items focusing on attitudes toward telerehabilitation.

Data analysis

Data analysis was conducted using IBM SPSS v29. Descriptive analysis was performed to assess qualitative aspects of the variables. Validation of the questionnaire included both construct validity and internal consistency. To identify the questionnaire’s factorial structure, Principal Components Analysis (PCA) with oblimin rotation was conducted to reveal latent factors and the total variance explained. Preliminary tests included the Bartlett’s Test of Sphericity and the Kaiser-Meyer-Olkin (KMO) measure of sampling adequacy. Reliability was assessed using Cronbach’s alpha to determine internal consistency. Chi-square tests for independence were used to evaluate correlations between variables according to predefined hypotheses [23]. If Chi-square assumptions were violated (threshold set at 20%), the Likelihood Ratio test was applied. Statistical significance was set at α = 0.05.

## Results

Construct validity of the questionnaire

The main 10-item questionnaire demonstrated moderately acceptable internal consistency (Cronbach’s alpha = 0.648), approximating the commonly accepted threshold of 0.7 [24]. Bartlett's Test of Sphericity was significant (Bartlett’s = 255.09, df = 45, p < 0.001), and the KMO Measure of Sampling Adequacy was above 0.6 (KMO = 0.614), indicating suitability for factor analysis. PCA revealed a three-factor solution, familiarization with technology, preferable mode of telerehabilitation, and willingness to attend telerehabilitation, with eigenvalues of 4.13 and 1.94, respectively, and explaining 53.96% of the total variance. Specifically, items Q1, Q2, Q3, Q5, and Q6 were grouped under the first factor; Q7, Q9, and Q10 under the second; and Q4 and Q8 under the third. The scree plot also supported a three-factor structure. Factor loadings ranged from 0.38 to 0.77, supporting the construct validity of the instrument.

Demographics/Background

The final sample consisted of 160 participants, after excluding 10 individuals from the initial 170 approached. Only complete cases were included, given the low missing data rate of 5.8%, which occurred completely at random. Participants’ demographic characteristics (e.g., gender, age, educational qualifications, and type of musculoskeletal condition) are presented in Table 1.

**Table 1:** Descriptive characteristics of participants with chronic musculoskeletal disorders.

Participant Characteristics	Categories	n (%)
Age Distribution	18-29 years	21 (13.1%)
	30-39 years	39 (24.4%)
	40-49 years	38 (23.8%)
	50-59 years	38 (23.8%)
	>60 years	24 (15%)
Gender	Female	95 (59.4%)
	Male	65 (40.6%)
Education Qualification	High School	71 (41.9%)
	University (BSc)	66 (41.3%)
	MSc / PhD	23 (14.4.%)
Profession	Public / Private Employees	119 (74.4%)
	Intellectual Workers	21 (13.2%)
	Manual Workers	3 (1.9%)
	Domestic Workers	10 (6.3%)
	Pensioners	7 (4.4%)
Frequency of Physical Activity	None	76 (47.5%)
	Once weekly	2 (1.3%)
	Twice weekly	47 (29.4%)
	Thrice weekly	17 (10.6%)
	More than 3 times weekly	18 ( 11.2%)
Medical Condition	Chronic Low Back Pain	56 (36.9%)
	Chronic Neck Pain	34 (21.3%)
	Chronic Back Pain (e.g., Scoliosis, Kyphosis)	3 (1.9%)
	Arthritis	14 (8.8%)
	Overuse Tendinopathies	50 (31.1%)
Familiarization with Technology / Internet	Good to Excellent	111 (69.4 3%)
	Moderate	34 (21.3%)
	Poor	15 (9.4%)
Preferred Devices	Smartphones	156 (46.6%)
	Laptops	114 (34%)
	Tablets	38 (11.3%)
	Smartwatches	27 ( 8.1%)
Perspective on Physiotherapy	Positive	143 (89.4%)
	Neutral	13 (8.1%)
	Ambiguous	4 (2.5%)
Perspective on Role of Exercise	Positive	156 (92.1%)
	Doubtful	4 (7.9%)

Participants’ attitudes towards telerehabilitation

In the main section of the questionnaire, responses regarding willingness to follow telerehabilitationwere as follows: 27.5% answered “maybe,” 21.3% “possible,” 20% “unlikely,” 15.6% “very likely,” and 15.6% “very unlikely.” Overall, only 36.9% of respondents expressed willingness to engage in telerehabilitation. For the management of their chronic musculoskeletal disorders, the majority of participants (62.4%) preferred face-to-face physiotherapy sessions, either at a clinic or at home, whereas 35.1% were comfortable with an initial face-to-face session followed by instructions delivered via phone or video call. Interestingly, over half (50.6%) reported that the COVID-19 pandemic served as the launching point for considering telerehabilitation as a viable treatment option. This suggests the pandemic played a substantial role in shaping patients’ openness to remote rehabilitation methods. A chi-square test of independence showed that there was significant association between the pandemic period and the thought of attending telerehabilitation, χ^2^_(4,160)_ = 37.7, p < 0.001), with a proportion of 74%-80% reporting willing to follow telerehabilitation due to the pandemic.

Cost also emerged as a notable factor influencing therapeutic preferences. Specifically, if telerehabilitation were offered at a lower cost, participants were more inclined to follow that option, χ^2^_(4, 160)_ = 10.2, p = 0.03). Table 2 presents the associations between willingness to engage in telerehabilitation and various influencing factors, highlighting both positive and negative contributors.

**Table 2:** Chi-square test of association between attitudes towards telerehabilitation and various variables in patients with chronic musculoskeletal disorders.

Variable	Pearson Chi-Square	p-value
Education level	38.7	0.007
Familiarization with technology	26.5	0.05
Pandemic period	37.7	0.001
Cost	10.2	0.03
Chronic musculoskeletal disorder	12.7	ns
Type of telerehabilitation	36.3	0.003
Tendency to look up medical information online	11.1	0.02
Perceived adherence	38.7	0.01

**Figure 1:**
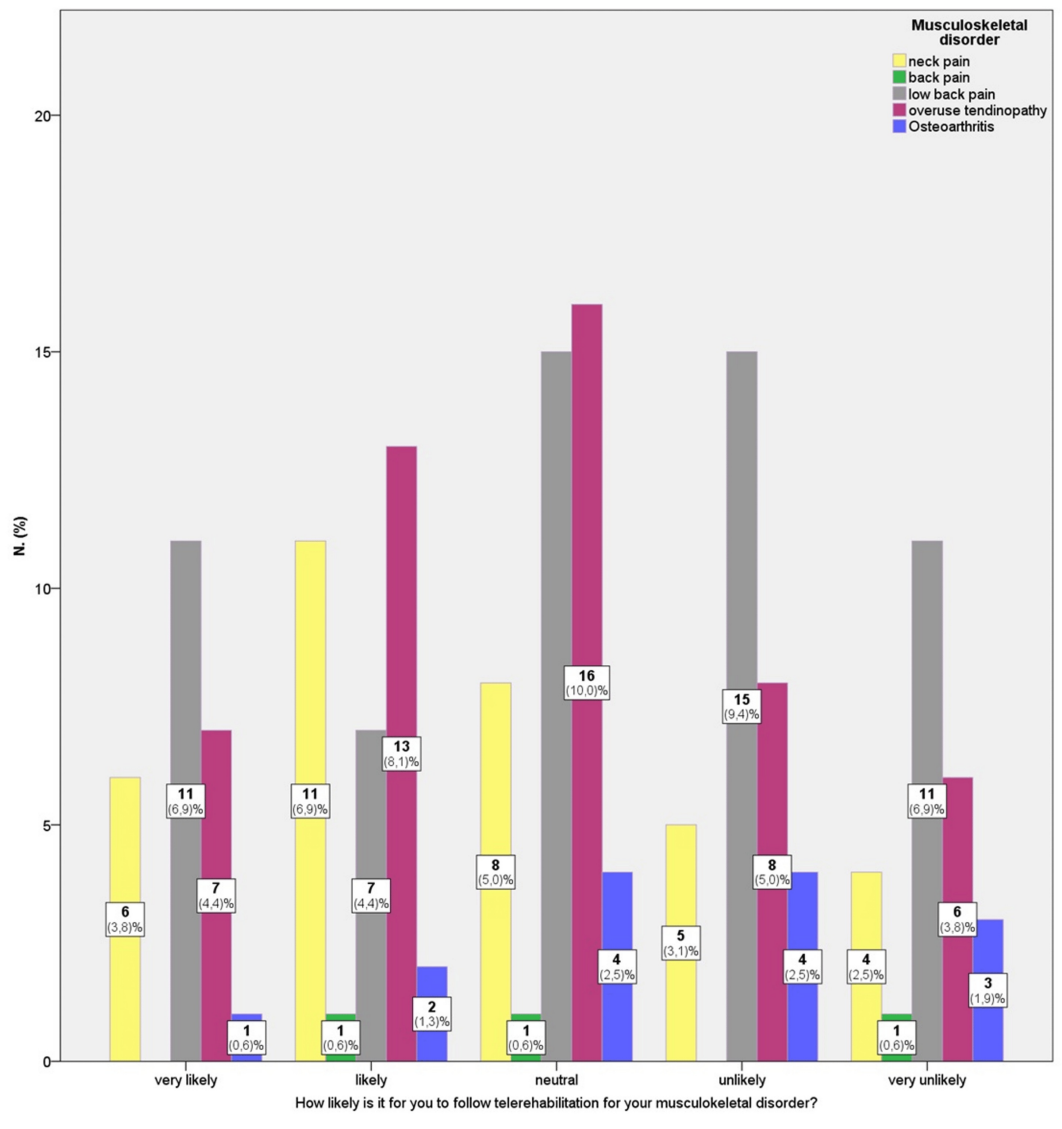
Willingness to use telerehabilitation according to participants’ disorder type (n = 160).

Neither the type of musculoskeletal disorder nor the time since diagnosis was found to be associated with participants’ willingness to engage in telerehabilitation (χ^2 ^_(16, 160)_=12.7, p = 0.7). Figure 1 illustrates the participants’ likelihood of accepting telerehabilitation based on their chronic musculoskeletal disorder. Overall, participants with low back pain (36.9%), followed by those with upper limb tendinopathies (31.3%) and neck pain (21.3%), appeared more receptive to telerehabilitation. In contrast, those with osteoarthritis (8.8%) and other back pain conditions such as osteoporosis, kyphosis, or scoliosis (1.9%) appeared more reserved.

A marginal correlation was observed between participants’ attitudes toward telerehabilitation and their level of technological familiarity (χ^2^_(16, 160)_ = 26.5, p = 0.048). Specifically, participants who expressed a positive inclination to attend telerehabilitation were more likely to report greater familiarity with technology (28%), whereas those who were more skeptical about telerehabilitation demonstrated poor technological familiarity (44%-48%).

**Figure 2:**
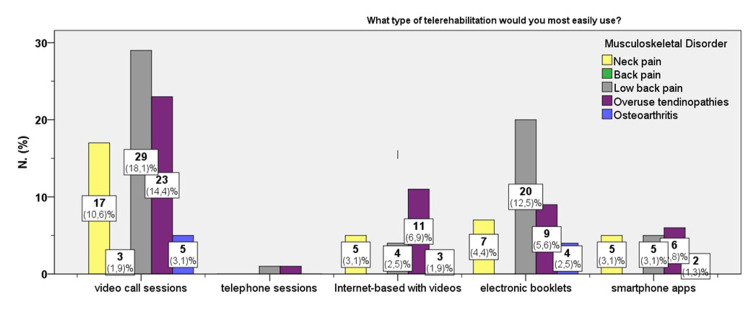
Participants’ preferences on the type of remote rehabilitation according to the diagnosis of chronic musculoskeletal disorder (n = 160).

Figure 2 presents participants’ preferred modes of remote rehabilitation based on their musculoskeletal condition. The figure indicates that across all groups, video-call sessions were the unanimously preferred mode, with the highest percentage reported among individuals with back pain.

A statistically significant correlation was also found between attitude toward telerehabilitation and the preferred type of remote rehabilitation (χ^2^_(16, 160)_ = 36.3, p=0.003). Contingency table analysis revealed that participants who were more likely to engage in telerehabilitation reported a preference for video-call sessions (43%-67%), while those who were less likely to participate tended to prefer physiotherapy booklets (52%).

Neither gender nor age was associated with participants’ attitudes toward telerehabilitation (p = ns). However, education level showed a statistically significant association with willingness to participate in telerehabilitation (χ^2^_(20,160) _= 38.3, p= 0.007). Participants with higher education levels were more open to telerehabilitation (University graduates: 50%-52%), whereas high school graduates were more hesitant (40%-68.8%). A marginally significant correlation was also found between participants’ attitudes toward telerehabilitation and their tendency to seek information online (χ^2^_(16,160)_ = 11.1, p= 0.02). Those with a positive attitude toward telerehabilitation were more likely to frequently look up information online related to their condition (56%-76%), while those less inclined to attend telerehabilitation reported not using the internet for this purpose (62.5%).Additionally, participants who considered themselves to have higher adherence to following medical guidelines reported greater willingness to pursue telerehabilitation (χ^2^_(20, 160)_ =38.7, p = 0.001).

## Discussion

The aim of the current study was to investigate and interpret individuals’ attitudes towards telerehabilitation for managing chronic musculoskeletal disorders. Based on the survey findings, less than half of the participants reported having a positive attitude towards telerehabilitation, reflecting a degree of ambivalence. The preferred mode of telerehabilitation was unanimously reported as video-based, and participants' willingness to engage in such an approach increased when initial sessions were conducted face-to-face. The main findings showed that willingness to undergo telerehabilitation was associated with a tendency to seek medical information online and with greater familiarity with technology. Interestingly, the type of musculoskeletal disorder did not appear to influence willingness to pursue telerehabilitation; however, this interpretation should be approached with caution due to low statistical power and variation in the sample. Participants with low back pain, neck pain, and upper limb tendinopathies appeared more receptive, whereas those with osteoarthritis and upper back pain were more hesitant.

Greater familiarity with technology and a higher education level were associated with a greater willingness to try self-management strategies via smart applications or internet/video-based sessions. This can be interpreted as higher education and technology literacy enhancing autonomy in the comprehension and application of the information and guidance provided. Relevant findings have been observed in the literature, where individuals with higher educational levels tend to seek health-related information online, while patients with lower health literacy often avoid seeking information beyond clinical encounters and face challenges in assuming the role of an “engaged patient” [25,26]. According to Baroni MP et al. [8], it is important to guide individuals who frequently gather health-related information from non-scientific online sources into becoming “digitally engaged patients,” as they are at risk of adopting non-evidence-based and often biased approaches. Online content is frequently not aligned with the best available evidence. Self-management-based treatment has been recommended for such patients [27]. Therefore, individuals should be safely directed to accredited telerehabilitation resources by healthcare professionals.

According to Fiani B et al. [10], the use of telerehabilitation increased during the COVID-19 pandemic among people with musculoskeletal pain. A study conducted in the United States reported that remote physiotherapy services during the pandemic were well accepted by patients, who not only participated actively but also found the results satisfactory and expressed willingness to continue with remote sessions post-pandemic [28]. The current findings align with this, as more than half of the survey respondents indicated that the COVID-19 period prompted them to consider telerehabilitation. Therefore, both community members and physiotherapists in public or private practice should be adequately informed to provide appropriate guidance to interested individuals. In the study by Braga LW et al. [29], the use of telerehabilitation over a three-year period during the pandemic was rated by most patients, caregivers, and healthcare professionals as an effective tool for managing various rehabilitation conditions.

In the study by Braga LW et al. [29], which evaluated new and follow-up patients’ satisfaction with telerehabilitation and their perception of its efficacy in an established network of rehabilitation hospitals post-pandemic, most patients described their remote consultations as being as good as or better than in-person visits. In the current study, participants reported having had a good previous experience with physiotherapy and, therefore, presented a positive attitude (~90%) toward benefiting again from physiotherapy for the management of their chronic condition. Additionally, around 46% believed that exercise could be a potential solution to their dysfunction. Approximately 62% were willing to visit a physiotherapist at an outpatient clinic or at home (~34%) for the initial sessions and then continue their care remotely. Video calls appeared to be the most preferred mode of remote contact (~50%), as participants felt safer with real-time interaction with the physiotherapist, particularly during the initial sessions. Fully guided videos were preferred by 15% of participants, followed by asynchronous guidance via smart applications (11.3%). Smartphones (97.5%) and PCs (71.3%) were the most frequently reported devices. Attitudes toward telerehabilitation were correlated with the perceived ease of using these devices. Consequently, booklets as a self-management option were typically selected by participants who were reluctant to engage in telerehabilitation. Therefore, a hybrid model of telerehabilitation, initiated with synchronous sessions and continued asynchronously, seems to be a more attractive and viable approach.

Cost was another encouraging factor for about 42% of participants, particularly if telerehabilitation was less expensive than in-person rehabilitation. Overall, lower educational level, concerns about receiving remote care without prior face-to-face guidance, and difficulty handling technological devices emerged as barriers to telerehabilitation. Thus, carefully designed strategies that emphasize the convenience and user-friendliness of digital platforms should be prioritized to better engage this population. Facilitators of positive attitudes toward telerehabilitation included higher education level, positive prior experiences with physiotherapy, belief in the benefits of exercise, and lower cost. These factors should all be considered when designing and promoting telerehabilitation services to increase participation and improve outcomes. In Greece, 61.3% of physiotherapists believe that telerehabilitation can be beneficial as a supplementary method of patient management and have already employed low-cost and easily accessible digital technologies, such as mobile phones and online meeting tools (e.g., Skype, Zoom), to support patient needs [30].

Study’ strengths and weaknesses

The final version of the survey questionnaire created for this purpose followed the steps suggested by Roopa S and Satya RM [22], in order to produce an instrument that would be useful, coherent, statistically scalable, and clearly reflective of the survey’s concept. The questionnaire was further reviewed by a group of experts to assess adequacy and scaling. Factorial analysis showed acceptable factor loading, and the grouping of items into the three factors, familiarization with technology, preferable mode of telerehabilitation, and willingness to attend telerehabilitation, contextually supported the labels assigned to these factors, indicating acceptable validity of the instrument. However, only modest reliability was observed, suggesting that the findings should be interpreted with caution. The study sample was balanced in terms of gender and included a relatively broad age group (18-65 years). Moreover, the educational level was medium to high (38% high school graduates, 41% university graduates), and the musculoskeletal conditions were primarily low back pain (37%) and upper/lower limb chronic injuries or tendinopathies (32%) lasting 3-6 months. Therefore, the sample was considered representative of the target population for assessing attitudes towards telerehabilitation. To avoid over-representation of technologically inclined individuals, the questionnaire was administered in paper format within the clinics.

Among the limitations of the study is that the questionnaire demonstrated only moderately acceptable internal consistency (approaching the 0.7 threshold), and thus the limited robustness of the instrument may have affected construct reliability. Although an appropriate power calculation was applied and the target sample size (n = 160) was achieved, it remains relatively small. Therefore, low power or sample variation may have influenced the results. Conducting the study with a larger sample could reduce variance and improve internal consistency. Additionally, participants were recruited regionally from Attica, the largest and most urbanized region in Greece. As such, the findings may not be generalizable to more rural areas across the country, where access to healthcare and health literacy levels may differ. The nature of survey-based research introduces inherent self-report bias, including social desirability and recall inaccuracy, which should be acknowledged. Finally, since all participants were already receiving physiotherapy, they may exhibit a positive bias toward the intervention compared to individuals without prior physiotherapy experience.

## Conclusions

Telerehabilitation culture is being constructed as a resource to supplement the rehabilitation process; however, the survey highlights that there are still barriers to overcome based on patients’ attitudes, as less than half appeared positive towards telerehabilitation. The tendency to seek medical information and familiarity with technology were associated with a more favorable attitude towards telerehabilitation. Interestingly, the type of chronic musculoskeletal disorder was not related to patients’ attitudes. A preference was observed for a flexible hybrid program that begins with in-person care and continues with a digital option. Easy access and low-cost video calls via smartphones or personal computers, along with the ability to integrate exercise under the supervision of a physiotherapist into daily routines, were considered important facilitators in adopting telerehabilitation.

Telerehabilitation has the potential to be established within a network of rehabilitation hospitals and clinics to support the self-management of individuals in need. Identifying the perceived needs, facilitators, and barriers to engaging in a telerehabilitation-supported self-management program, from the perspective of patients with chronic musculoskeletal disorders, is crucial. Therefore, the insights gained from this study may provide valuable input for developing improved infrastructure to support physiotherapists in delivering telerehabilitation, as well as for creating user-friendly, clinically relevant, and tailored solutions to guide self-management for chronic musculoskeletal conditions. A promising opportunity for integrating telerehabilitation into routine musculoskeletal care emerges; however, future longitudinal studies are needed to validate these findings, given their limited generalizability.

## Appendices

Appendix 1 

**Table 3:** Outline of the questionnaire content.

	Content - (No. of relevant questions) (Sample items)
Preliminary part (16 items)	Demographics (5)
	Patients’ knowledge of pathology (2)
	Patients’ awareness of self-management strategies (1)
	Previous physiotherapy experience (3)
	Tendency to adhere to exercise/advice (1)
	Lifestyle perspective on physical activity/exercise (2)
	Intention to look up medical information on the internet (1)
Specific main part (10 items)	Familiarization with technological means – Telerehabilitation (10)
	Familiarization with technological means/internet/smartphone apps (4) (i.e. How familiar are you with technological devices?)
	Preferable technological device (i.e. What technological device do you most frequently use daily?)
	Preferable mode of telerehabilitation (i.e. What type of telerehabilitation would you use more easily?)
	Patients’ willingness to follow telerehabilitation (i.e. Would you use telerehabilitation to help you handle your pain problem?)
	Potential adherence to rehabilitation (i.e. How dedicated will you be in following exercise via telerehabilitation at home?)
	Whether COVID-19 affected view on telerehabilitation (i.e. Has COVID-19 affected your attitude towards telerehabilitation?)
	Cost/Preference (i.e. Since telerehabilitation costs less, would that affect your decision to use it?)

Appendix 2 

**Table 4:** Complete survey questionnaire.

Survey Questionnaire		
General Part		
Α. Demographics		
N.	Question	Value
1	Gender	Male, Female
2	Age (years)	
3	Educational level	Lower than high School, High School graduate, Bachelor Graduate, MSc Graduate, PhD Graduate
4	Frequency of exercise/ physical activity per week	None, Once, Twice, 3 times >3 times
5	Work field	Public/Private servants, Manual Workers (i.e. plumber, builder etc.), Intellectual workers (i.e. lawyer, teacher, researcher etc.), Domestic housework, Pensioners, other
B. Chronic Musculoskeletal Disorder		
6	Area of musculoskeletal pain	Neck pain, Back pain (scoliosis, kyphosis), Low back pain, Overuse tendinopathies, Arthritis, Other
7	How long have you been diagnosed with your chronic musculoskeletal disorder?	3-6 months, 6-12 months, 1-5 years, 5-10 years, ≥10 years
8	How do you manage your disorder? *You can choose more than one option	Physiotherapy, Injection, Acupuncture, Medication, Alternative methods (e.g., homeopathy), Surgery, Other
9	Physiotherapy has helped me in the past manage my pain problem	Strongly agree, Agree, Neutral, Disagree, Strongly disagree
10	How often do you visit your physiotherapist for your musculoskeletal disorder?	Daily, Weekly, Monthly, Annually, Every 2-5 years
11	My therapy outcome depends mostly on my physiotherapist	Strongly agree, Agree, Neutral, Disagree, Strongly disagree
12	Do you tend to follow your physiotherapist’s advice?	I follow it regularly, Often, Occasionally, Only when in pain, I do not follow it
13	Do you think an exercise program is necessary for your pain?	Strongly agree, Agree, Neutral, Disagree, Strongly disagree
14	Being physically active will help my pain	Strongly agree, Agree, Neutral, Disagree, Strongly disagree
15	Are you aware of ways to self-manage your chronic pain?	Strongly agree, Agree, Neutral, Disagree, Strongly disagree
16	How often do you search for medical information on the internet?	A great deal, Much, Somewhat, Little, Never
Specific Part		
C. Familiarization With Technology		
17	How familiar are you with technological devices (e.g., PC, tablet, smartphone)?	Very poor, Below average, Average, Above average, Excellent
18	What technological device do you most frequently use daily?	Smartphone, PC, Tablet, Smartwatch, Other
19	How often do you use the internet?	A great deal, Much, Somewhat, Little, Never
20	How important is a smartphone in your daily life?	Very important, Important, Moderately important, Slightly important, Not important at all
21	How often do you use smartphone applications in your daily life?	Always, Daily, Weekly, Monthly, Never
D. Attitudes Towards Rehabilitation		
22	Would you use telerehabilitation to help manage your musculoskeletal pain?	Very likely, Likely, Neutral, Unlikely, Very unlikely
23	COVID-19 affected your attitude towards telerehabilitation	Strongly agree, Agree, Neutral, Disagree, Strongly disagree
24	Would you follow exercise via telerehabilitation at home?	Very likely, Likely, Neutral, Unlikely, Very unlikely
25	Since telerehabilitation costs less, would that affect your decision to use it?	Strongly agree, Agree, Neutral, Disagree, Strongly disagree
26	What type of telerehabilitation would you find easiest to use?	Video calls, Phone calls, Internet videos, Smartphone applications, Electronic booklets/web-based material
